# Supplementary figures and images for: Spatiotemporal dynamics of ecosystem services in response to climate variability in Maze National Park and its environs, southwestern Ethiopia
Source: PLoS One. 2024 Jul 26;19(7):e0307931. doi: 10.1371/journal.pone.0307931 (PMC11280226; doi:10.1371/journal.pone.0307931)

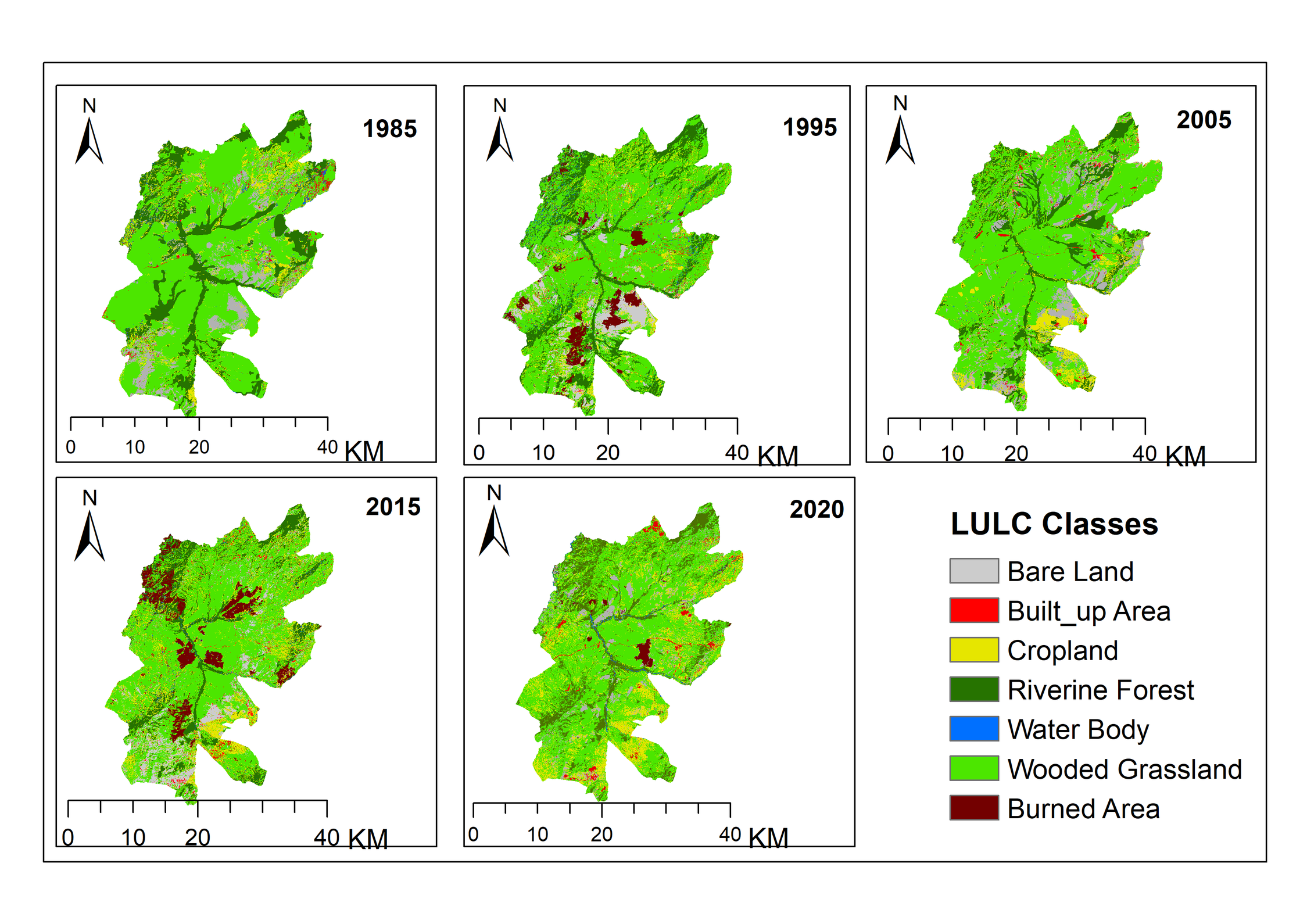

Supplement: S1 Fig — (TIF) [file pone.0307931.s001.tif]

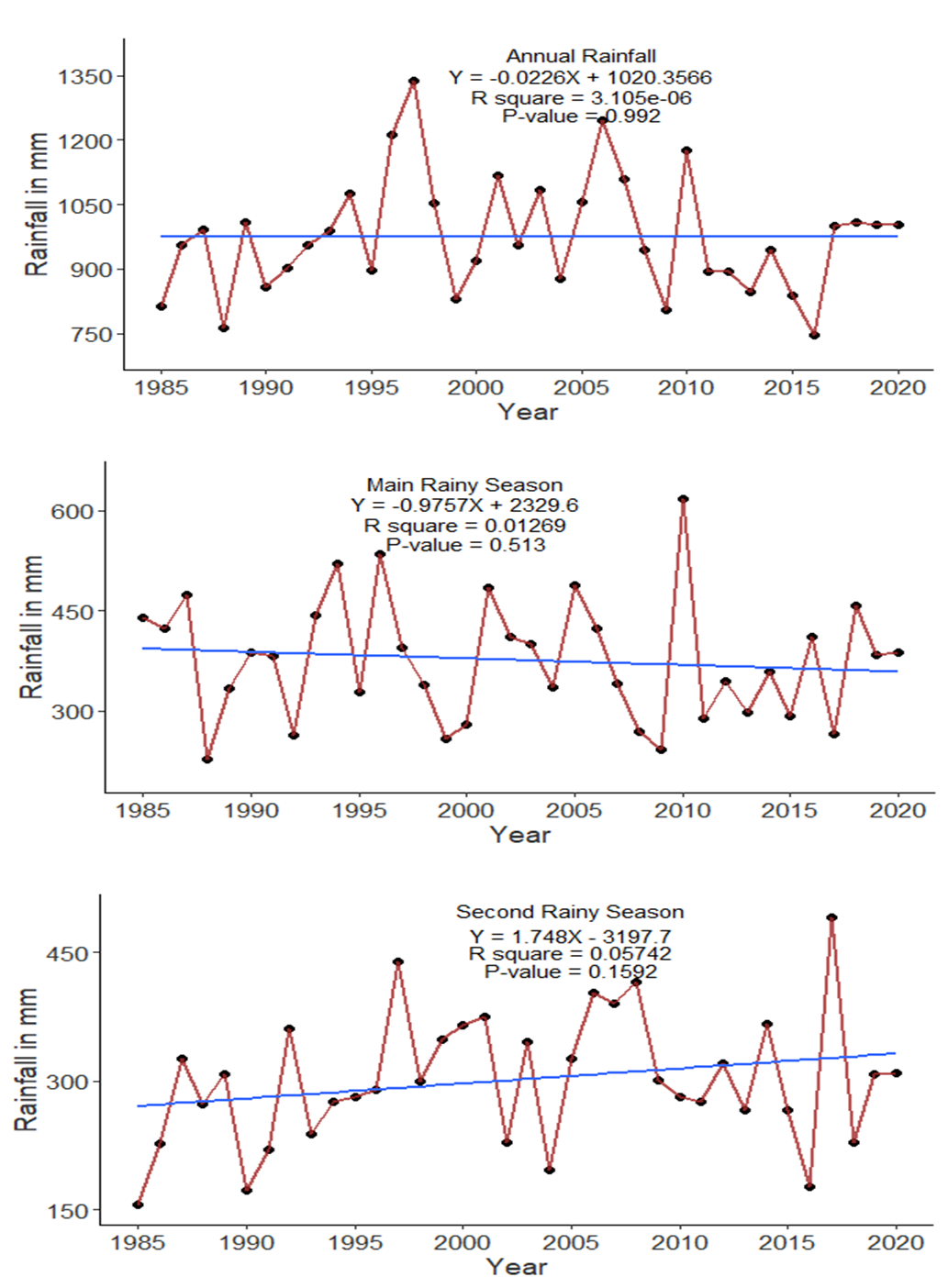

Supplement: S2 Fig — (TIF) [file pone.0307931.s002.tif]

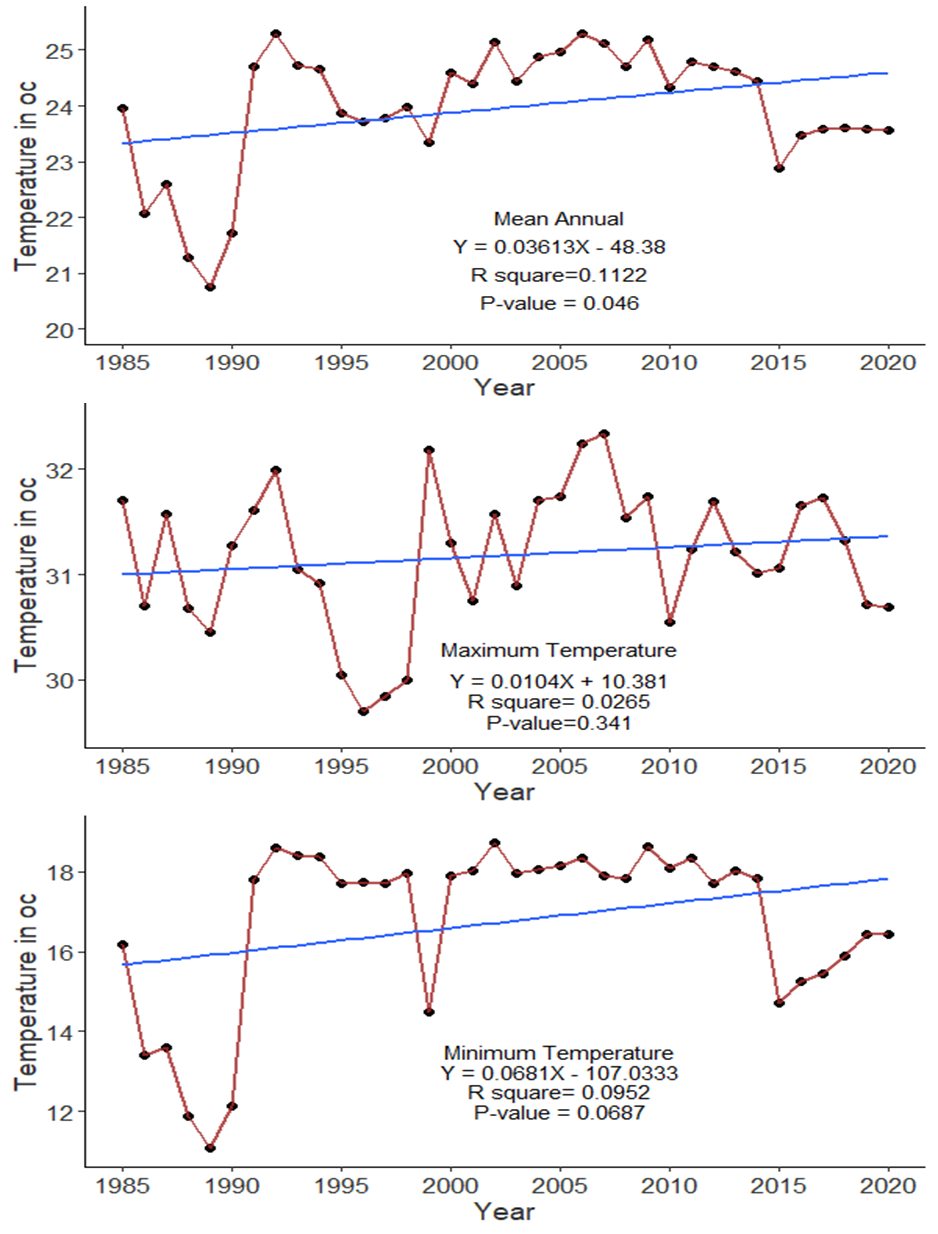

Supplement: S3 Fig — (TIF) [file pone.0307931.s003.tif]
