# Supplementary material for: Spatiotemporal dynamics of ecosystem services in response to climate variability in Maze National Park and its environs, southwestern Ethiopia
Source: PLoS One. 2024 Jul 26;19(7):e0307931. doi: 10.1371/journal.pone.0307931 (PMC11280226; doi:10.1371/journal.pone.0307931)
Supplement: S1 Table — (DOCX) [file pone.0307931.s004.docx]

Satellite images used for LULC classification and ecosystem services valuation (Simeon and Wana,2024) and this study

| Imagery Type | Path/Row | Pixel Size(m) | Bands Used | Acquisition Date | Source |
| --- | --- | --- | --- | --- | --- |
| Landsat TM | 169/56 | 30*30 | 1-5 and 7 | 01/09/1985 | USGS |
| Landsat TM | 169/56 | 30*30 | 1-5 and 7 | 01/20/1995 | USGS |
| Landsat ETM^+^ | 169/56 | 30*30 | 1-5 and 7 | 01/24/2005 | USGS |
| Landsat OLI | 169/56 | 30*30 | 2-7 | 01/28/2015 | USGS |
| Landsat OLI | 169/56 | 30*30 | 2-7 | 12/11/2020 | USGS |
