# Supplementary material for: Spatiotemporal dynamics of ecosystem services in response to climate variability in Maze National Park and its environs, southwestern Ethiopia
Source: PLoS One. 2024 Jul 26;19(7):e0307931. doi: 10.1371/journal.pone.0307931 (PMC11280226; doi:10.1371/journal.pone.0307931)
Supplement: S2 Table — (DOCX) [file pone.0307931.s005.docx]

S2 Table: Accuracy assessment result of the classified images

| LULC Classes | Accuracy (%) | | | | | | | | | | | | | | |
| --- | --- | --- | --- | --- | --- | --- | --- | --- | --- | --- | --- | --- | --- | --- | --- |
|  | 1985 | | | 1995 | | | 2005 | | | 2015 | | | 2020 | | |
|  | PA | UA | Kappa hat | PA | UA | Kappa hat | PA | UA | Kappa hat | PA | UA | Kappa hat | PA | UA | Kappa hat |
| Bare Land | 88.65 | 84.82 | 0.83 | 96.91 | 80 | 0.79 | 92.47 | 98.23 | 0.98 | 78.74 | 90 | 0.90 | 94.82 | 94.69 | 0.94 |
| Built-up Area | 55.50 | 93.22 | 0.93 | 100 | 80 | 0.8 | 73.67 | 96.61 | 0.97 | 100 | 80 | 0.80 | 77.59 | 91.53 | 0.91 |
| Cropland | 70.50 | 97.33 | 0.97 | 100 | 100 | 1.00 | 98.52 | 89.33 | 0.88 | 100 | 90 | 0.89 | 86.81 | 84.00 | 0.81 |
| Riverine Forest | 84.40 | 88.98 | 0.86 | 100 | 100 | 1.00 | 84.47 | 80.51 | 0.77 | 95.40 | 100 | 1.00 | 87.63 | 98.81 | 0.99 |
| Water Body | 42.49 | 86.54 | 0.86 | 100 | 100 | 1.00 | 24.09 | 76.92 | 0.76 | 29.98 | 100 | 1.00 | 65.63 | 76.92 | 0.77 |
| Wooded Grassland | 97.16 | 88.36 | 0.73 | 99.67 | 96.67 | 0.92 | 97.96 | 95.99 | 0.90 | 96.55 | 93.33 | 0.86 | 95.57 | 91.63 | 0.80 |
| Burned Area | - | - | - | 58.06 | 100 | 1.00 | - | - | - | 100 | 100 | 1.00 | 63.58 | 94.20 | 0.94 |
| Overall Accuracy | 88.93 | | | 96.11 | | | 93.03 | | | 93.93 | | | 91.57 | | |
| Kappa Coefficient | 0.81 | | | 0.94 | | | 0.88 | | | 0.91 | | | 0.86 | | |
