# Supplementary material for: Spatiotemporal dynamics of ecosystem services in response to climate variability in Maze National Park and its environs, southwestern Ethiopia
Source: PLoS One. 2024 Jul 26;19(7):e0307931. doi: 10.1371/journal.pone.0307931 (PMC11280226; doi:10.1371/journal.pone.0307931)
Supplement: Attachment — Submitted filename: Response to Reviewers.docx [file pone.0307931.s011.docx]

**Responses for Revisions of Manuscript**

Manuscript Number: PONE-D-24-05650

Title: Spatiotemporal dynamics of ecosystem services in response to climate variability in Maze national park and its environs, southwestern Ethiopia

Journal: PLOSONE

Dear editor and reviewers,

Thank you for your valuable time and effort in reviewing our manuscript and providing insightful comments and suggestions. We have carefully considered all your comments, and almost all of them have been accepted and incorporated into the revised manuscript. To strengthen the literature review, highlight the novelty of our work, and enrich the discussion, we have reviewed an additional 13 articles. Changes made in the manuscript are marked using Track changes, and the newly added references are highlighted in blue in the text and reference section. We have provided point-to-point responses to your comments and highlighted them in blue as follows:

Thank you for your review

**Journal Requirements:**

1. When submitting your revision, we need you to address these additional requirements. Please ensure that your manuscript meets PLOS ONE's style requirements, including those for file naming. The PLOS ONE style templates can be found at https://journals.plos.org/plosone/s/file?id=wjVg/PLOSOne_formatting_sample_main_body.pdf andhttps://journals.plos.org/plosone/s/file?id=ba62/PLOSOne_formatting_sample_title_authors_affiliations.pdf.

**Response:** Thank you for the suggestion. We have adjusted our manuscript according to the journal’s requirements.

2. Please note that PLOS ONE has specific guidelines on code sharing for submissions in which author-generated code underpins the findings in the manuscript. In these cases, all author-generated code must be made available without restrictions upon publication of the work. Please review our guidelines at https://journals.plos.org/plosone/s/materials-and-software-sharing#loc-sharing-code and ensure that your code is shared in a way that follows best practice and facilitates reproducibility and reuse.

**Response:** Thank you. We did not generate any new code for this study. However, we utilized pre-developed codes for the Mann-Kendall test, Sen's slope estimator, ITA tests, and for generating temperature and rainfall trend graphs in R statistical software.

3. We note that the grant information you provided in the ‘Funding Information’ and ‘Financial Disclosure’ sections do not match. When you resubmit, please ensure that you provide the correct grant numbers for the awards you received for your study in the ‘Funding Information’ section.

**Response:** Thank you for your comment. We have now provided the grant information in the 'Funding Information' section of the online submission system.

4. We note that your Data Availability Statement is currently as follows: [All relevant data are within the manuscript and its Supporting Information files]. Please confirm at this time whether or not your submission contains all raw data required to replicate the results of your study. Authors must share the “minimal data set” for their submission. PLOS defines the minimal data set to consist of the data required to replicate all study findings reported in the article, as well as related metadata and methods (https://journals.plos.org/plosone/s/data-availability#loc-minimal-data-set-definition).

For example, authors should submit the following data:

- The values behind the means, standard deviations and other measures reported;

- The values used to build graphs;

- The points extracted from images for analysis.

Authors do not need to submit their entire data set if only a portion of the data was used in the reported study. If your submission does not contain these data, please either upload them as Supporting Information files or deposit them to a stable, public repository and provide us with the relevant URLs, DOIs, or accession numbers. For a list of recommended repositories, please see https://journals.plos.org/plosone/s/recommended-repositories.

If there are ethical or legal restrictions on sharing a de-identified data set, please explain them in detail (e.g., data contain potentially sensitive information, data are owned by a third-party organization, etc.) and who has imposed them (e.g., an ethics committee). Please also provide contact information for a data access committee, ethics committee, or other institutional body to which data requests may be sent. If data are owned by a third party, please indicate how others may request data access.

**Response:** Thank you. We have uploaded data used for rainfall and temperature variability analysis, trend tests (Tables and graphs), and points generated from images for spatial analysis in supporting information files.

5. Please upload a copy of S1 Figure, S2 Figure, S3 Figure and S1 Table to which you refer in your text on page 33. Please amend the file type to 'Supporting Information'. If the Supplementary file is no longer to be included as part of the submission please remove all reference to it within the text.

**Response:** Thank you for the comment. Copies of S1 Figure, S2 Figure, S3 Figure, S1 Table, and S2 Table have been uploaded in supporting information files.

**Responses for Reviewers' comments:**

**Reviewer #1**

Abstract:

**Comment 1:** The abstract summarizes the study effectively, but lacks specific quantitative findings.

**Response**: Thank you very much for the comment. Due to the word limit, we have now included some major quantitative findings in the abstract section, lines 39 to 48.

Introduction:

The introduction provides a comprehensive background on the importance of ecosystem services and the impact of climate variability. It effectively highlights the relevance of the study by emphasizing the lack of empirical research on this topic in Ethiopia. The introduction outlines the specific objectives of the study, aiming to fill this research gap, by focusing on the spatiotemporal impacts of climate variability on key ecosystem services in MzNP. However, there are some critical points to be improved:

**Comment 2:** The references are appropriate but could be expanded to include more recent studies on similar topics globally to strengthen the argument for the study’s novelty.

**Response:** Thank you for this comment. In order to strengthen the literature review, we have included additional four (4) recent studies in the introduction section in page 1 paragraph 1, lines 60 to 72 and page 2 paragraph 2, lines 102 to 111.

**Comment 3:** The identification of research gap is well articulated, but it could benefit from more detailed discussion on why previous studies were insufficient.

**Response:** Thank you very much. We addressed the research gap in the second paragraph of page 2, lines 98 to 111, by comparing our study with previous research on ecosystem good and services. Previous studies primarily focused on the impacts of LULC changes on ecosystem services, overlooking the effects of climate variability and lacking empirical analysis of these variables.

**Comment 4:** The text should consistently distinguish between different types of ecosystem services to enhance clarity.

**Response:** Thank you for this comment. Different types of ecosystem services were discussed according to the Millennium Ecosystem Assessment (MEA) classification in page 1, paragraph 1, lines 60 to 64.

**Comment 5:** It is necessary to use consistent terms for climate-related phenomena and impacts. For example, “climate change” and “climate variability” are used interchangeably but have distinct meanings.

**Response:** Thank you for the comment. While climate change and variability are distinct terms, the impacts of climate change often manifest through climate variability. However, we have now focused more on climate variability and also provided their distinctions in introduction section page 1, paragraph1, lines 67 to 72.

**Comment 6:** To give some examples of the mechanisms by which climate change affects ecosystem services, will provide a more comprehensive understanding of the linkages and processes involved.

**Response:** Thank you very much. We have discussed the mechanisms by which climate change and variability affect ecosystem services on page 1, paragraph 2, lines 78 to 85. It is stated as follows: “The changing climate, evidenced by climate variability, affects the provision of ecosystem services and the well-being of people who rely on these services[12] . Ethiopia, like many other African countries, is very susceptible to the negative impacts of climate change and variability due to its low adaptive capacity[13]. In Ethiopia, climate change, characterized by high temperature, reduced rainfall, and increased rainfall variability and its manifestations in extreme weather conditions have negatively affected ecosystem services such as food production, groundwater availability, and soil organic matter and soil quality[14].”

**Comment 7:** The aims are quite broad, and the authors don’t provide specific research questions that guide the research. Defined aims and question would provide a sharper focus for the study.

**Response:** Thank you for the comments as well. In the introduction section on page 2, paragraph 2, lines 115 to 120, we have now included the specific objectives addressed in this study.

Methods:

**Comment 8**: The methods section is detailed and describes the study area, however, discuss specific characteristics of MzNP that make it a relevant case study for examining the impacts of climate variability on ecosystem services, such as its biodiversity, ecosystem types, or known climate vulnerabilities.

**Response:** Thank you for this comment. We have included more detailed descriptions of the study area, the ecosystem services obtained from there, and the major factors contributing to the decline of these services on page 3, paragraph 2, lines 130 to 132, and page 4, paragraph 1, lines 151 to 156.

**Comment 9:** The methods for collecting climate data are clear, but the explanation for ecosystem services data is vague. I suggest providing a more detailed explanation of how ecosystem services data were measured or quantified. I suggest specifying the indicators used, the data sources, and the methods of data collection.

**Response**: Thank you for the suggestion. The valuation of ecosystem services was based on Costanza et al. (1997) valuation method considering the contribution of each LULC classes for multiple ecosystem services. We applied benefit transfer method to the valuation of ecosystem services using recently developed ecosystem services value coefficients of Ecosystem Services Valuation Database (ESVD) (de Groot et al., 2020). The detailed explanation about ecosystem service value estimation from LULC classes using benefit transfer method was explained in our previous study (Simeon and Wana, 2024, pages 4 and 5) from which ecosystem service values of the study area in 1985, 2005, and 2020 were taken and similar procedure was followed for estimation of 1995 and 2015 values (page 5, paragraph 1 and 2, lines 180 to196).

**Comment 10:** The use of the MK trend test, Sen’s slope estimator, and ITA are appropriate for analyzing climate trends. The use of Pearson correlation and partial correlation to evaluate relationships among variables is also well-justified. However, while the description of the study area is thorough, including more specific reasons for choosing MzNP would strengthen the rationale. Finally, there should be a discussion on the assumptions underlying the statistical methods and how they were addressed, this will provide robustness to the methodology and ensure the validity of the results.

**Response:** Thank you very much. We have discussed the assumptions underlying the statistical methods employed in this study and how they were addressed in the methods section. The assumptions for the Mann-Kendall (Mk) trend test and Sen’s slope estimator were considered due to their insensitivity to outliers and lack of distribution assumptions. However, they may contain some error if autocorrelation exists in the time series data. Thus, autocorrelation was tested by calculating the autocorrelation coefficient at lag-1. To address this issue, the modified Mann-Kendall method was used to correct serially autocorrelated data with a significant lag-1 autocorrelation coefficient using the variance correction method in R statistical software (Page 7 paragraph 1 lines 230 to 238). Response: The ITA method is valid regardless of the sample size, serial correlation structure of the time series, and non-normal probability distribution of the data. Therefore, no underlying assumptions were employed (page 8, paragraph 3, lines 262 to 266). For Pearson and partial correlation analysis, since the distribution of the data affects parametric tests, histograms and normality tests were employed to determine whether the distribution of the data was normal. Non-normally distributed data were then transformed using Log10 data transformation techniques (page 9 paragraph 2 lines 283 to 286).

Results:

**Comment 11:** The results are presented clearly, with the findings from the MK test, ITA and correlation analyses well-organized. The results show significant trends in climate variables and their correlations with ecosystem services. However, visual aids (e.g, graphs, charts) could enhance the clarity and impact of the findings. While the results are clear, the interpretation could be more nuanced. I suggest a time series graph showing temperature and rainfall trends over the study period would provide a clearer picture of the data.

**Response:** Thank you for the suggestion. Time series graphs of the mean annual, main rainfall season, and second rainfall season, as well as the mean annual, maximum, and minimum temperatures, have now been attached as supporting information files S2 and S3.

Discussion:

**Comment 12:** The discussion section interprets the results in the context of existing literature, emphasizing the implications for ecosystem services and climate adaptation strategies. It addresses the study’s limitations and suggests areas for future research. However, while the results are clear, the interpretation could be more nuanced. I suggest discussing potential reasons for the observed trends and correlations would add depth. This could provide a broader context for results. I suggest discussing potential ecological or social factors that might explain the observed trends and correlations. For example, consider the role of land-use changes, conservation policies, or socio-economic conditions in the region.

**Response:** Thank you for the comment. We have incorporated the potential reasons for the observed variabilities, trends and correlations. For instance, we explained the reasons for high rainfall variability (page 12, paragraph 2, lines 375 to 377), temperature variability (page 15, paragraph 1, lines 444 to 446), the correlation between minimum and maximum temperature and raw materials (page 21, paragraph 2, lines 595 to 601), and the correlation between minimum and maximum temperature and climate regulation services (page 21, paragraph 3, lines 608 to 614).

**Comment 13:** There is limited comparison with findings from other regions. This will help place the results in a broader context and highlight similarities or differences in climate impacts on ecosystem services across different regions.

**Response:** Thank you very much. Additional previous studies from different continents and regions are included for comparison with our findings. These are presented on page 12, paragraph 1, lines 369 to 371; page 13, paragraph 2, lines 405 to 408; page 16, paragraph 1, lines 455 to 457; page 21, paragraph 1, lines 598 to 601; and page 21, paragraph 2, lines 608 to 611.

**Comment 14:** In general, the discussion is insightful but could benefit from deeper analysis. I suggest exploring the mechanisms driving the observed trends and correlations in greater detail. For example, discuss how specific climatic factors, such as seasonal rainfall patterns or temperature extreme, affect different ecosystem services like water provisioning, biodiversity, or carbon sequestration.

**Response:** Thank you for the comment. We have discussed how rainfall, temperature, altitude, and NDVI distribution affect ecosystem services on page 19, paragraph 2, lines 539 to 542; page 21, paragraph 2, lines 593 to 598; and page 21, paragraph 3, lines 608 to 609.

**Comment 15:** On the other hand, the practical implications are mentioned but not elaborated upon. I suggest providing concrete recommendations for local stakeholders, such as adaptive management practices, conservation strategies, or community engagement approaches to mitigate the impacts of climate variability. I suggest providing specific recommendations for future research methodologies and potential study areas.

**Response:** Thank you for the suggestion. We have added more specific practical implications on page 24, paragraph 2, lines 673 to 677. Additionally, we have included specific valuation methods and potential study areas on page 24, paragraph 2, lines 684 to 688.

**Comment 16:** I suggest integrating more recent studies and broader geographical contexts to enhance the discussion. This will help situate the study within the larger body of research on climate variability and ecosystem services.

**Response:** Thank you for this comment. Recent studies at the continental level (Africa) and from other continents have been incorporated into the discussion section on page 13, paragraph 2, lines 405 to 408; page 16, paragraph 1, lines 455 to 457; page 21, paragraph 1, lines 598 to 601; and page 21, paragraph 2, lines 609 to 611.

**Reviewer #2**

GENERAL COMMENTS

In the article titled “Spatiotemporal Dynamics of Ecosystem Services in Response to Climate Variability in Maze National Park and its Environs, Southwestern Ethiopia”, the authors evaluated the impacts of temperature and rainfall variability on selected key provisioning (food production, water supply, raw material) and regulatory ecosystem services (climate regulation) in Maze National Park.

In this regard, I comment that the introduction is too general, it only mentions the trends or response patterns of ecosystem services to the effect of climate change or climate variability, which are not necessarily synonyms. It is necessary to provide greater context of the effect on ecosystem services, explain response mechanisms, processes involved, etc. Furthermore, it is not clearly justified why the four key services were selected. Services are not defined. For food production and raw material services, it would be appropriate to clearly define which crops or raw materials are being considered within the context of Maze Nation Park. It is recommended to show more forcefully how this case study contributes to the global literature, not just the country or the national park, on the response of ecosystem services to climate variability when considering a wide range of data (years, precipitation, temperature). and analysis techniques (CV, RAI, MK, Sen slope, ITA, Pearson and partial Correlation Analysis).

**Response:** Thank you for this comment. We have revised the introduction to highlight the specific impacts of climate variability on ecosystem services. Although climate change and variability are distinct terms, climate change impacts are manifested through climate variability. However, we have now focused more on climate variability and also provided their distinctions in introduction section page 1, paragraph1, lines 67 to 72. The selection of key ecosystem services is mentioned in the methods section on page 4, paragraph 3, lines 172 to 176. We highlighted the contribution of our study to global literature on page 24, paragraph 1, lines 668 to 671. Regarding definitions of ecosystem services, we provided descriptions in Table 1 on page 5.

In the methods, there is an imbalance in describing how the data was processed. For the climatic variables there is very fine detail in how they were processed and the tests used to show trends and variability are clearly indicated. On the other hand, for services, it is only indicated that LULC maps are used and transformed into four ecosystem services. It is not explained which land use or cover land is linked to which service.

**Response:** Thank you for the comment. The ecosystem services values for the study years were estimated from LULC maps using the benefit transfer method and the Ecosystem Valuation Database (ESVD) value coefficients. The 1985, 2005, and 2020 values were directly taken from our previous study (Simeon and Wana, 2024), which was not published at the time of submitting this manuscript to PLOS ONE. The 1995 and 2015 ecosystem services values were estimated using the same procedure. Detailed methods for ecosystem services valuation are presented in our previous work (Simeon and Wana, 2024, pages 4 - 5).

In the results and discussion, unexpected findings are not discussed, for example (page 12, paragraph 2, lines 3 and 4) “…. Unexpectedly, November rainfall alone showed a statistically significant increase at 99% confidence interval…”. There is no integrated discussion of the spatiotemporal dynamics of the four ecosystem services (food production, water supply, raw material, and climate regulation) in response to the four variables related to climate and productivity (precipitation, temperature, evapotranspiration, NDVI) and its variability (mean annual, main and second season, maximum, minimum). Mainly, the trends of the results are compared with other studies, the results from different sections are rarely integrated. The strengths and limitations of the study are not discussed. Some limitations are mentioned in the conclusions.

**Response:** Thank you very much. We have discussed the unexpected monotonic increase of November rainfall on page 13, paragraph 2, lines 391 to 396 as follows: “This might be explained by changes in the timing of the rainy seasons over the years, particularly since 2003, due to the impacts of climate change and variability. However, this requires further investigation.” The spatial variability of climate variables and NDVI and their correlation with ecosystem services were analyzed and discussed using the mean annual values of precipitation, temperature, and NDVI from pages 16 to 19. The temporal dynamics of ecosystem services in response to temperature (maximum and minimum), rainfall (annual and seasonal), and evapotranspiration were discussed from pages 19 to 23. In the conclusion, we have addressed the major limitations of this study and provided recommendations for future research (page 24, paragraph 2, lines 681 to 687).

SPECIFIC COMMENTS BY SECTION

Keywords

**Comment 1:** It is suggested that the words in this section be different from the words in the title.

**Response:** Thank you for the comment. We have revised the keywords based on the suggestion.

Introduction

**Comment 2:** Page 1 Paragraph 1 Lines 1,2, 7 and 8. The opening and closing phrases are very similar in the information they provide. It is suggested to improve the information and messages.

**Comment 3:** P1P1L4. Missing dot (.) after [4]

**Response for comment 2 and 3:** Thank you for the comment. We have revised and corrected the statements on page 1, paragraph1, lines 58 to 67.

**Comment 4:** P1P2L3-7 When it mentions “In Ethiopia, climate change and its manifestations in rainfall variability…... and human health conditions “There are different climatic conditions and different services, it is a very general sentence “…. have negatively affected ecosystem services…”, no mechanisms or processes are mentioned that support the pattern described. Also, when they mention “crop production” what crops? Do all crops in Ethiopia respond the same to precipitation or temperature? Furthermore, “human health conditions” is not an ecosystem service.

**Comment 5:** P1P4L3 Missing period (.) after [16-19]

**Response for comment 4 and 5**: Thank you very much. We have made corrections based on the aforementioned comments and presented them on page 1, paragraph 2, lines 78 to 85, and page 2, paragraph 2, lines 95 to 97 as follows: “In Ethiopia, climate change, characterized by high temperature, reduced rainfall, and increased rainfall variability and its manifestations in extreme weather conditions have negatively affected ecosystem services such as food production, groundwater availability, and soil organic matter and soil quality[14].” And, “…. However, the supply of these vital ecosystem goods and services are dwindling due to climate change and variability-induced changes, such as recurrent droughts, and other factors like increasing intensities of illegal forest fire[18]’

**Comment 6:** P2P4 It is not clearly justified why those four key services were selected (food production, water supply, raw material, and climate regulation services). No further context is given.

**Response:** Thank you for this comment. The reasons for selecting these four key ecosystem services are justified in the materials and methods section on page 4, paragraph 3, lines 172 to 176 stated as follows: “Based on Focus group discussions (FGDs) and interviews with local community, agricultural extension agents, and park staff, four ecosystem goods and services, such as food production, water supply, raw material provision, and climate regulatory services (Table 1) that the community perceives as major benefits[33] and directly impacted by climate change and variability were identified.”

Materials and methods

**Comment 7:** P3P1L4 What does kebeles mean? Are they districts?

**Response:** Thank you very much. *Kebeles* (the smallest administrative units in Ethiopia) are defined on page 3, paragraph 2, lines 129 to 130.

**Comment 8:** Figure 1. Scale bars are missing from maps B and C, as well as the compass rose. On map A, the 17 neighboring districts of the national park cannot be distinguished.

**Response:** Thank you for the comment. We have added scale bars and north arrows to maps B and C, and overlaid the 17 surrounding districts of the park on Map A of Figure 1.

**Comment 9:** P4P2L1-2 Did the discussion with local community, agricultural extension agents, and park workers take place in workshops or interviews?

**Response:** Thank you very much. The identification of major ecosystem services involved discussions with the local community, agricultural extension agents, and park workers through focus group discussions and key informant interviews (page 4, paragraph 3, lines 172 to 176). The sample size, sampling technique, and other detailed procedures are presented in our previous study (Simeon and Wana, 2024, pages 6 and 7).

**Comment 10**: P4P2L2 What crops are considered in food production? What is raw material provision within the context of the MzNP?

**Response:** Thank you very much. We have defined the key ecosystem services in Table 1 on page 5. For clarity, food production in Maze National Park and its surrounding areas refers to crop production (including cereals and tuber crops) and the collection of wild fruits from the park region. Raw materials include the collection of firewood, charcoal, thatching grasses, cutting trees for fences and house construction, and the collection of fodder (grasses for domestic animals).

Table 1:Description of key ecosystem services

| Ecosystem Service | Description | Sources |
| --- | --- | --- |
| Food production | Production crops, nuts, fruits by hunting, gathering, subsistence farming or fishing. | [2,36] |
| Water supply | Provisioning of water by watersheds for drinking, irrigation and other domestic use | [36,37] |
| Raw materials | The production of lumber, fuel wood, fodder/pasture, charcoal, thatching grass | [36,37] |
| Climate regulation | Regulation of local temperature, precipitation | [2,36] |

**Comment 11:** P4P2L5 What LULC was associated with food production? What LULC was associated with raw material? Which LULC was associated with climate regulatory services? It is also important to detail why these LULCs are associated with services within the local context. How ecosystem service values were estimated from the LULC maps? Only one land use or cover type were used for service or several?

**Response**: Thank you. The valuation of ecosystem services was based on Costanza et al. (1997) valuation method, considering the contribution of each LULC type to multiple ecosystem services. The valuation applied the benefit transfer method using the recently developed ecosystem services value coefficients of the Ecosystem Services Valuation Database (ESVD) (de Groot et al., 2020). A detailed explanation of ecosystem service value estimation from LULC classes using the benefit transfer method is presented in our previous study (Simeon and Wana, 2024, pages 4 and 5), from which the ecosystem service values for the study area in 1985, 2005, and 2020 were taken. A similar procedure was followed for estimating the 1995 and 2015 values (page 5 paragraphs 1 and 2, lines 180 to 196).

**Comment 12:** P4P2L13 What is the resolution of the satellite images used to do the supervised classification? Is the resolution higher or lower than the climate variability grid data?

**Response:** Thank you very much. The satellite images used for the supervised classification were Landsat images with a 30-meter spatial resolution. The grid climate data has a spatial resolution of 4km by 4km. For this study, we employed averaged temperature and rainfall data generated from seven grid points for temporal temperature and rainfall trend analysis. These averaged values were then interpolated with a similar cell size to create a continuous surface for spatial analysis.

**Comment 13:** P5P5L1-2 Add space between rainfall (mm) and series (mm)

**Comment 14:** P6P1L1-2 Add space between series (mm)

**Comment 15:** P9P1L4-5 Define if it is very strong or strong “…and very strong positive/negative correlation (≥±.8). strong positive/negative correlation (≥±.8) …”

**Response of comments 13, 14, and 15**: Thank you for the comment. We have added a space between rainfall (mm), series (mm), and series (mm) on page 6, paragraph 4, lines 219 to 222. And we have defined the scales of Pearson’s correlation values on page 10, paragraph 1, lines 304 to 307 as follows: “very weak positive/negative correlation (0 < |r| < ±0.2); weak positive/negative correlation (±0.20 ≤ |r| < ±0.4), moderate positive/negative correlation (± 0.40 ≤ |r| < ±0.6); strong positive/negative correlation (±0.60 ≤ |r| < ± 0.8), and very strong positive/negative correlation (≥±.8) following[57] and [58].”

**Comment 16:** P10P1L Since the study site has two distinct rainfall seasons and rainfall affects the NDVI values (season of greatest greenness and growth), what period of the year (months or days) did the Landsat images cover to obtain the average NDVI?

**Response:** Thank you. While it is generally preferable to use Landsat satellite images from the rainy seasons to better capture NDVI, for the sake of consistency in our correlation analysis between key ecosystem services and NDVI values, we opted for satellite images taken during similar months and dates. We used Landsat images with minimal cloud cover for both NDVI and ecosystem service values estimation. Except for the 2020 image, which was acquired in December, the images for 1985, 1995, 2005, and 2015 were all acquired in January. Detailed descriptions of the satellite images are uploaded in supporting information S1 Table.

Results and discussion

**Comment 17:** In general, for this entire section it is suggested to be consistent in the order in which the results of the services are presented (food production, water supply, raw material, and climate regulation services), because it usually varies between tables and between figures. It is suggested to present them according to the order in which they are mentioned from the introduction.

**Response**: Thank you for the suggestion. We have made corrections to ensure consistency throughout the manuscript regarding the order of key ecosystem services: food production, water supply, raw material, and climate regulation.

**Comment 18:** P12P2L1-3 In Table 2, the data do not match the decreasing trend of April and June, as well as mean annual, and the main rainy season rainfall.

**Response:** Thank you for the comment. The negative Sen's slope values for April, June, mean annual, and main rainy season rainfall presented in Table 3 indicate a decreasing trend of rainfall during the study periods.

**Comment 19:** P12P3L3 in “Mk” the “K” is capitalized

**Comment 20:** P13P1L1-3 The information “…..According to [46], if …… 1:1 straight line….” It is presented in the materials and methods (P7P4L8-11). It is suggested to leave the explanation in only one of the two sections.

**Response of comments 19 and 20**: Thank you for the suggestion. We have capitalized “K” on page 14, paragraph 2, line 412, and removed the repeated explanation on page 14, paragraph 3, lines 416 to 419.

**Comment 21:** P15P3L3. The paragraph mentions the correlation between mean evapotranspiration and ecosystem services, but the evapotranspiration map is not shown in Figure 8, there is no description of the spatial variability of this variable, as is done for precipitation and temperature. Furthermore, correlation results are not shown in Table 6. What is the reason?

**Response**: Thank you for pointing that out. Evapotranspiration was mistakenly included in page 16, paragraph 3, line 473, under spatial correlation analysis between climate variables and ecosystem services. We actually used evapotranspiration to assess its temporal impact on ecosystem services in addition to major determining factors (rainfall and temperature), using different correlation tests (partial correlation method). That's why it is not included in the spatial analysis results (Fig. 8 and Table 7).

**Comment 22:** Figure 9 So that the NDVI maps are visually comparable and easy to distinguish spatial and temporal variations, it is suggested that the color legend consider the most negative value (-0.37) and the most positive value (0.74), or failing that, that the color legend ranges from -1 to 1. For example, the color orange covers values from -0.37 to 0, while blue covers values from 0.50 to 0.74. Finally, the range of NDVI value per year is described in detail in the text (P16P1L3-5).

**Response:** Thank you for the feedback. The most negative value (-0.37) and the most positive value (0.74) are observed in different years (2005 and 1995, respectively) to display NDVI values in each year. The values of NDVI are displayed in Fig. 9 using stretched values represented by colors. The color scale ranges from orange (representing the lowest value) to dark blue (representing the highest value), indicating a continuous range of values rather than categorical or classified values.

**Comment 23:** P18P1L1-9 Considering that the value of ecosystem services was derived from LULC maps and that the NDVI (a measure of greenness) that is highly correlated with NPP, plant cover, and ecosystem productivity (P17P1L1-2), was it obvious the high correlation shown in the results of Table 6?

**Response**: Thank you very much. The correlation result presented in Table 7 indicates a statistically significant but weak spatial correlation between NDVI and ecosystem services. This suggests that while spatial variability of NDVI has an impact on ecosystem services, there are other important determinants affecting the distribution of ecosystem services as well.

**Comment 24:** P19P1L15-7 It is suggested to discuss the result “…On the other hand, the climate regulation service in the study area exhibited a negative correlation with the minimum and maximum temperature…” as is done with other services.

**Response:** We appreciate the feedback. We have included discussions on the correlation between climate regulation and minimum and maximum temperatures, as well as additional relationships with LULC changes. This is presented in page 21 paragraph 2 lines 602 to 614.

**Comment 25:** P19P3L1-2 It is suggested to present the information on raw material services from when it is first presented in the introduction “…includes the production of lumber, fuel, or fodder that are extracted as raw materials.”

**Response:** Thank you for the suggestion. According to Costanza (1997), raw material services encompass the production of lumber, fuelwood, and fodder. In our local context and in this study, raw materials include the collection of firewood, charcoal, fodder or pasture, thatching grasses, lumbering, and cutting of trees for house and fence building. Additionally, we have defined the key ecosystem services included in this study in Table 1 on page 5.

**Comment 26:** P20P2 The results of the evapotranspiration and services correlations are very far from where they were first mentioned (page 15).

**Response:** Thank you. Potential evapotranspiration was employed to illustrate only the temporal dynamics of key ecosystem services in response to annual and seasonal evapotranspiration. The correlation results are discussed in detail on page 22, paragraph 2, lines 635 to 651 and presented in Table 9.

Supporting information

**Comment 27:** S2 and S3 Figure. It is suggested to add the P value (significance level) to the functional relationships between years and rainfall and temperature, not just the R square value.

**Response:** Thank you for your feedback. P values have been included in the temperature and rainfall trend graphs in figures S2 and S3.

**Note**: The detailed explanation of the land use land cover classifications and ecosystem services valuation for the years 1985, 2005, and 2020 is provided in our previous study (Simeon and Wana, 2024), which was not published at the time of submitting this manuscript to the PLOSONE journal. We followed a similar procedure for the valuation of services in 1995 and 2015. During this revision, we cited our previously published paper for a detailed explanation of ecosystem services valuation.

Sincerely Yours

Mestewat Simeon, Corresponding author
